# Optical-Fiber Power Meter Comparison between NIST and KRISS*

**DOI:** 10.6028/jres.117.019

**Published:** 2012-12-21

**Authors:** I. Vayshenker, S. K. Kim, K. Hong, D.-H. Lee, D. J. Livigni, X. Li, J. H. Lehman

**Affiliations:** 1 National Institute of Standards and Technology, Boulder, CO 80305, USA; 2 Center for Photometry and Radiometry, Division of Physical Metrology, Korea Research Institute of Standards and Science, Daejeon 305–340, Republic of Korea

**Keywords:** international comparison, optical fiber, optical power

## Abstract

We describe the results of a comparison of reference standards between the National Institute of Standards and Technology (NIST-USA) and Korea Research Institute of Standards and Science (KRISS-R.O. Korea) for optical fiber-based power measurements at wavelengths of 1302 nm and 1546 nm. We compare the laboratories’ reference standards by means of a temperature-controlled optical trap detector. Measurement results showed the largest difference of less than 2.5 parts in 10^3^, which is within the combined standard (*k*=1) uncertainty for the two laboratories’ reference standards.

## 1. Introduction

In our previous work [1–8], we reported the results of international comparisons of reference standards used in the calibration of optical fiber power meters (OFPMs). Those reports describe the results that were obtained by use of open laser beams [1,4,6] and optical fiber cables [2–8] at nominal wavelengths of 1310 nm and 1550 nm. In this paper, the reference standards maintained by the two national laboratories (NIST and KRISS) were compared at wavelengths of 1302 nm and 1546 nm by launching optical power from a reference optical fiber.

For OFPM measurements, the primary standards of both NIST [9] and KRISS [10] are cryogenic radiometers that have standard uncertainties of 2 parts in 10^4^ (*k*=1). Typically, reference standards are calibrated against the primary standards by use of open (free-field) collimated beams, but are generally used with divergent beams of laser light exiting an optical fiber. Most primary standards are designed to be used with open beams rather than divergent beams from an optical fiber. Thus, a transfer standard that is insensitive to beam geometries (either collimated or divergent beam) is a very important tool for comparing reference standards.

## 2. Transfer Standard

**Fig. 1:**
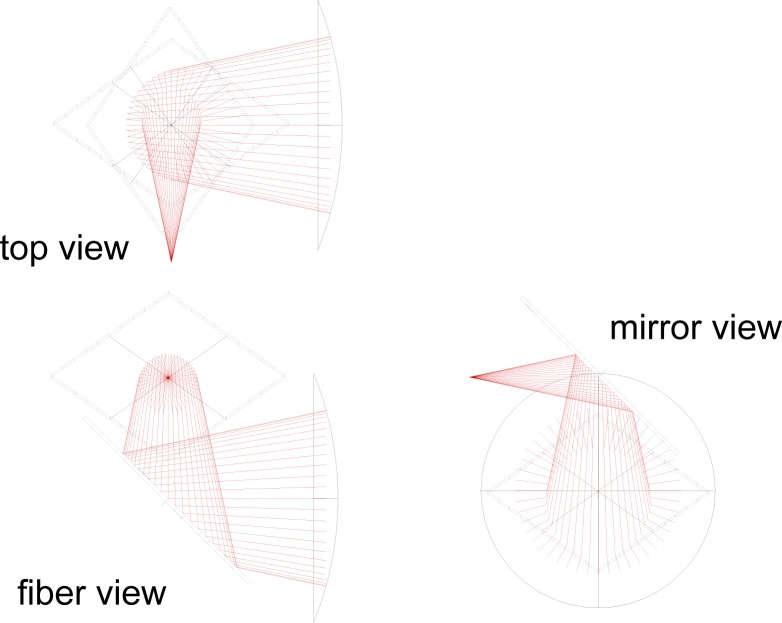
Schematic representation of the photodiodes, concave mirror, and fiber output depicted in three views.

For this comparison we used a transfer standard designed and built by NIST [11]. The transfer standard depicted in Fig. 1 is an optical-trap detector that consists of two germanium photodiodes and a spherical mirror. The two Ge photodiodes are 10 mm in diameter and the concave aluminum mirror has a 15 mm diameter and 40 mm focal length, and uniform coating of magnesium fluoride. The two photodiodes are oriented relative to the entrance aperture so that the principal ray of incident radiation strikes each diode once at a 45° angle of incidence and then reflects from the concave mirror back onto the photodiodes in reverse order. The photodiodes and mirror are enclosed in a thermoelectrically cooled environment. It has been shown in [12] that such a configuration provides a uniform response over the field of view and therefore requires no correction for beam geometry. The Ge-trap detector was calibrated at both national laboratories against their reference standards. The same lasers, operating at wavelengths of 1302 nm and 1546 nm, and same optical fiber cable with fiber connectors with physical contact (FC/PC) were used by both laboratories, which employed a direct-substitution method for their measurements. This transfer standard is also referred as Device Under Test (DUT).

## 3. Measurement System

**Fig. 2:**
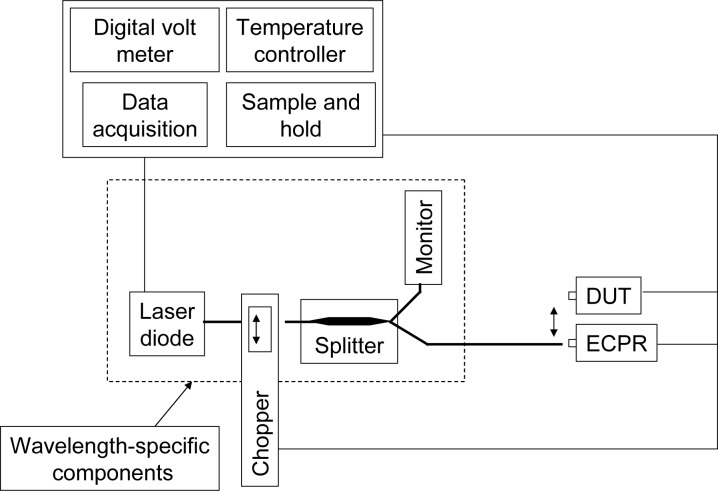
Measurement system that utilizes fiber-pigtailed laser diode sources at wavelengths of 1302 nm and 1546 nm.

The NIST and KRISS measurement systems are very similar; therefore in this section we describe the common measurement system used by both laboratories. The NIST measurement system is depicted in Fig. 2; it consists of fiber-pigtailed laser diode sources at wavelengths of 1302 nm and 1546 nm (all center wavelengths in this paper are based on refractive index in vacuum), a reference optical fiber cable, and a positioning stage (see double-headed arrow) for comparing the reference and transfer (DUT) standards. The NIST measurement system is described in more detail in [13] and KRISS’s measurement system is described in [14]. Both laboratories’ reference standards are electrically calibrated pyroelectric radiometers (ECPRs) that have been previously calibrated against the primary standards. The ECPR consists of a thermal detector that is covered with gold black coating. The response of the ECPR does not depend on the wavelength of the incident radiation over the wavelength region of 1300 nm to 1550 nm [15].

## 4. Results of the Comparison

The NIST and KRISS reference standards were compared at an optical power of approximately 100 µW (−10 dBm). The standard uncertainties for the optical power measurements were evaluated in accordance with ISO document standards [16].

Both laboratories used the same reference optical fiber cable. At NIST six measurement runs were taken with relative standard deviation of 0.9 × 10^−3^ at a wavelength of 1302 nm and relative standard deviation of 0.1 × 10^−3^ at a wavelength of 1546 nm. At KRISS, six measurement runs were taken with a relative standard deviation of 0.1 × 10^−3^ at 1302 nm and eight measurement runs were taken with a relative standard deviation of 0.2 × 10^−3^ at 1546 nm. The results of the comparison are given in Table 1.

At 1302 nm the relative difference between the NIST and KRISS results was 2.5 parts in 10^3^, and at 1546 nm the relative difference was 1 part in 10^3^ (the plus sign for both relative differences indicates that the KRISS reference standard read lower than that of NIST). The NIST standard uncertainty was 2 parts in 10^3^ at 1302 nm and 2.5 parts in 10^3^ at 1546 nm, while that of KRISS was 3.5 parts in 10^3^ at 1302 nm and 3.6 parts in 10^3^ at 1546 nm.

Table 1 provides values of relative combined standard uncertainty for NIST and KRISS. These values are calculated by taking a square root of the sum of the squares of each laboratory standard uncertainty. The observed interlaboratory differences (0.25 % at 1302 nm and 0.10 % at 1546 nm) are less than the relative combined standard (*k*=1) uncertainties for the laboratories’ reference standards.

## 5. Conclusion

The comparison results demonstrate that the largest difference between NIST and KRISS measurements is well within the combined standard (*k*=1) uncertainty for the laboratories’ reference standards. Therefore, this optical-fiber power meter comparison shows good agreement between NIST and KRISS reference standards. The purpose of this work is to verify a consistency in measurements of optical fiber power in the area of optical telecommunications.

## Figures and Tables

**Table 1:** Results of NIST and KRISS comparison

Source wavelength (nm)	Difference (%)	KRISS standard uncertainty (%)	NIST standard uncertainty (%)	Combined standard uncertainty (%)
1302	0.25	0.35	0.20	0.40
1546	0.10	0.36	0.25	0.43
